# An endogenous human peptide derived from α_1_-antitrypsin as a novel pharmacological inhibitor of *Bordetella pertussis* toxin

**DOI:** 10.1007/s00210-025-04744-1

**Published:** 2025-11-17

**Authors:** Stefanie Lietz, Lena-Marie Sokolowski, Alexander Beyschlag, Helena Rosenau, Annika Siewert, Armando A. Rodríguez Alfonso, Nico Preising, Ludger Ständker, Sebastian Wiese, Janet Köhler, Gilbert Weidinger, Jan Münch, Arto T. Pulliainen, Katharina Ernst, Holger Barth

**Affiliations:** 1 Institute of Experimental and Clinical Pharmacology, Toxicology and Pharmacology of Natural Products, Ulm University Medical Center, 89081 Ulm, Germany; 2 Core Facility Functional Peptidomics, Ulm University Medical Center, 89081 Ulm, Germany; 3 Core Unit Mass Spectrometry and Proteomics, Ulm University Medical Center, 89081 Ulm, Germany; 4 Institute of Biochemistry and Molecular Biology, Ulm University, 89081 Ulm, Germany; 5 Institute of Molecular Virology, Ulm University Medical Center, 89081 Ulm, Germany; 6 Institute of Biomedicine, University of Turku, 20520 Turku, Finland

**Keywords:** *Bordetella pertussis*, Pertussis toxin, Toxin inhibitor, Endogenous proteins, Endogenous peptides, α_1_-antitrypsin

## Abstract

Pertussis, also known as whooping cough, is a highly infectious respiratory disease caused by the bacterium *Bordetella pertussis*. The bacterial virulence factor, pertussis toxin (PT), is associated with the manifestation of the characteristic symptoms of pertussis and the severe form of this disease. Increasing case numbers and the lack of treatment options highlight the need to develop novel pharmacological strategies, e.g., the generation of specific PT inhibitors. Recently, we identified the endogenous human protein α_1_-antitrypsin (α_1_AT) as an inhibitor of PT from a screening of a human hemofiltrate protein/peptide library. In the present work, we tested an in-house α_1_AT peptide bank to identify an α_1_AT region with anti-PT activity. Then, we compared the sequences of the positive hits from the peptide bank with all known α_1_AT fragments in the hemofiltrate samples to find new active peptides. In total, 36 peptides were tested for their PT inhibition, leading to the identification of an endogenous α_1_AT fragment, α_1_AT HF, derived from hemofiltrate with anti-PT activity. This peptide had no toxic effects on HeLa cells and in vivo on zebrafish embryos, rendering it an attractive lead compound for further evaluation to treat pertussis in the future.

**Supplementary Information:**

The online version contains supplementary material available at 10.1007/s00210-025-04744-1.

## Introduction

Recently, increasing case numbers for the vaccine-preventable, highly infectious respiratory disease pertussis *alias* whooping cough, caused by the bacterium *Bordetella (B.) pertussis*, have been reported by the European Centre for Disease Prevention and Control (ECDC). With over 32,000 reported cases in the EU/EEA countries in the first 3 months of 2024, annual pre-COVID-19-pandemic case numbers of pertussis were almost exceeded, e.g., 41,026 cases in 2016 and 34,468 cases in 2019 (European Centre for Disease Prevention and Control 2024). As such, despite the extensive vaccination programs, pertussis is on the rise, leading to high levels (Locht and Antoine 2021; Yeung et al. 2017). Especially, vulnerable groups, including infants younger than 3 months who are too young to be vaccinated, suffer from severe pertussis (Mattoo and Cherry 2005). Severe pertussis disease is accompanied by characteristic complications that can require hospitalization, including long-lasting paroxysmal cough, leukocytosis, pneumonia, encephalopathy, seizures, or apnea which can also ultimately cause death (Mattoo and Cherry 2005; Surridge et al. 2007). Moreover, severe cases of pertussis are associated with the important virulence factor pertussis toxin (PT) secreted by *B. pertussis* (Mattoo and Cherry 2005; Scanlon et al. 2019).

PT is an AB-type protein toxin with ADP-ribosyltransferase (ART) activity. As an AB_5_-holotoxin with a 1:1:1:2:1 stoichiometry, PT consists of an enzymatically active A-subunit (PTS1) and a pentameric binding B-subunit (PTS2-5) (Stein et al. 1994; Tamura et al. 1982; Weiss et al. 1993). After binding to sialic acid conjugates on target cells facilitated by the B-subunit, PT is internalized into endosomes via receptor-mediated endocytosis (Armstrong et al. 1988; Witvliet et al. 1989). Intracellularly, PT follows a retrograde transport route from the endosomes to the Golgi apparatus and finally travels to the endoplasmic reticulum (ER) (el Bayâ et al. 1997; Plaut and Carbonetti 2008). The ATP stores within the ER facilitate the attachment of ATP to the central pore of the B-pentamer, which triggers the disassembly of the PT holotoxin and the release of PTS1 (Burns and Manclark 1986; Hazes et al. 1996). Detached PTS1 is thermally unstable, and its disordered/unfolded state is recognized by the ER associated degradation pathway (ERAD) and discharged from the ER into the cytosol (Banerjee et al. 2016; Pande et al. 2006). This translocation of PTS1 into the cytosol of the target cell occurs with the help of host cell chaperones (Ernst et al. 2018, 2021; Kellner et al. 2019, 2021). Here, it modifies its target proteins, the α-subunit of inhibitory G proteins (Gαi) associated with G protein-coupled receptors (GPCRs) (Bokoch et al. 1983). This modification prevents the Gαi-mediated inhibition of the adenylate cyclase, enhancing the production of cyclic AMP (cAMP) (Katada and Ui 1982). Finally, this leads to an altered signaling transduction, which in turn causes the development of specific symptoms associated with the toxin-mediated disease.

Since there are no direct therapeutic options for pertussis, targeted pharmacological approaches, such as inhibitors of PT, are very appealing and urgently required to combat and manage the disease. Here, a huge potential for the discovery of novel treatment strategies might be in the human peptidome (Bosso et al. 2018). Recently, we identified α_1_-antitrypsin (α_1_AT) by screening a human protein/peptide library as an endogenous inhibitor of PT activity (Lietz et al. 2024b). Moreover, α_1_AT was shown to be a PAN toxin inhibitor, as it additionally inhibited *Clostridium (C.) botulinum* C2 toxin, *Corynebacterium (C.) diphtheriae* diphtheria toxin, and a *Bacillus (B.) anthracis* fusion toxin (Lietz et al. 2024a). Therefore, α_1_AT appears as a promising candidate for further pharmacological analyses against toxin-mediated diseases, also in a clinical setting. Since α_1_AT-containing drugs are already approved and clinically applied for the treatment of α_1_AT deficiency, the repurposing of such drugs for treatment of pertussis could be addressed. In particular, our first studies using the α_1_AT-containing drug Prolastin® for treatment of *B. pertussis* infection in the infant mouse model for pertussis were promising (Lietz et al. 2024b). Here, the hallmark of pertussis, leukocytosis, was reduced when *B. pertussis*-infected mice were treated with α_1_AT in direct comparison to control mice that were also infected with *B. pertussis* but not treated with α_1_AT (Lietz et al. 2024b). As α_1_AT-containing drugs are produced from humans, enough donor material is required. A recombinant production of α_1_AT would be independent from human donors and would allow specific modifications to optimize α_1_AT for treatment of pertussis. In this context, the identification of the amino acid region within α_1_AT that harbors the anti-PT activity would be important. To this end, we generated various peptides based on two approaches, partially covering the endogenous sequence of α_1_AT. Here, we show that a specific region of α_1_AT that is located in the middle, positions 240–281 (UniProt ID P01009 precursor), bears anti-PT activity. The shortest generated α_1_AT fragment with anti-PT activity within this region has the position 252–262 (UniProt ID P01009 precursor). This α_1_AT fragment was found in an endogenous protein/peptide library originating from human hemofiltrate and had no adverse effects on cell viability and no toxic effects in vivo on zebrafish embryos, rendering it a promising lead compound for novel peptide-based therapeutics against pertussis.

## Results

### Overview of approaches to generate α_1_AT peptides with anti-PT activity

Prompted by our latest findings that describe endogenous α_1_AT as an inhibitor of *B. pertussis*, PT, *C. botulinum* C2 toxin, *C. diphtheriae* diphtheria toxin, and a *B. anthracis* fusion toxin (Lietz et al. 2024a, b), we were interested in the amino acids of the α_1_AT sequence that are responsible for the inhibitory mode of action. Primarily, we aimed to identify peptides derived from α_1_AT that inhibit PT. In this regard, two different approaches were applied. First, we searched our in-house endogenous peptide bank derived from α_1_AT fragments, comprising the already published α_1_AT fragment virus-inhibitory peptide (VIRIP), corresponding to the C-proximal region (precursor 377–396) of endogenous α_1_AT (Münch et al. 2007), as well as other 34 in-house peptides (F1–F5 and 32–88, see Table 1) covering several α_1_AT sequence regions. Most of these peptides were previously generated in a project investigating α_1_AT as an inhibitor of SARS-CoV-2 virus cell entry by directly interacting with the cell surface protease TMPRSS2 (Wettstein et al. 2021). Second, since we previously identified α_1_AT as an inhibitor of PT based on a screening of an in-house human hemofiltrate library (Lietz et al. 2024b), we searched the library for endogenous α_1_AT fragments (α_1_AT HF, one peptide). The sequences of the positive hits from testing the in-house α_1_AT fragments were compared (multiple sequence alignment) to our hemofiltrate peptide database to find fragments within the α_1_AT region covered by the positive hits (Supplementary Fig. 1). Within this region, we found only one peptide sequence NIQHCKKLSSW (252–262, α_1_AT HF, see Table 1), which was synthesized for further evaluation. In total, 36 synthetic peptides derived from α_1_AT were identified for subsequent functional analyses (Supplementary Fig. 2).

**Table 1:** Overview of all α_1_AT peptides investigated in this study. All tested peptides derived from α_1_AT are listed with their name, sequence, origin, and whether they possess anti-PT activity

Peptide name	Sequence (positions in α_1_AT precursor sequence 1–418, UniProt ID P01009)	Origin	Significant anti-PT activity
VIRIP	LEAIPMSIPPEVKFNKPFVF (377–396)	In-house (published)	No
F1	GKVVNPTQK (410–418)	In-house	No
F2	PLFMGKVVNPTQK (406–418)	In-house	No
F3 = 78	LMIEQNTKSPLFMGKVVNPTQK (397–418)	In-house	No
F4	SVLGQLGITK (325–334)	In-house	No
F5	VHKAVLTID (357–365)	In-house	No
32	LVNYIFFKGKWER (208–220)	In-house	No
36	YIFFKGKWER (211–220)	In-house	No
41	SIPPEVKFNKPFVFLMI (383–399)	In-house	No
42	HCKKLSSWVLLMKYLGNATAIFFLP (255–279)	In-house	Yes
44	HCKKLSSWVLLMKYLGNATAIFFLPDE (255–281)	In-house	No
49	LKLSKAVHKAVLTIDEKGTEAAGAMFLEAIPM (351–382)	In-house	No
50	IFFKGKWER (212–220)	In-house	No
51	ALVNYIFFKGKWER (207–220)	In-house	No
53	APLKLSKAVHKAVLTIDEKGTEAAGAMFLEAI (349–380)	In-house	No
61	VKVPMMKRLGMFNIQHCKKLSSWVLLMKYLGNATAIF (240–276)	In-house	No
62	CKKLSSWVLLMKYLGNATAIFF (256–277)	In-house	No
63	KKLSSWVLLMKYLGNATAIFF (257–277)	In-house	No
64	KRLGMFNIQHCKKLSSWVLLMKYLGNATAIF (246–276)	In-house	Yes
65	HCKKLSSWVLLMKYLGNATAIFFLPD (255–280)	In-house	No
66	LKLSKAVHKAVLTIDEKGTEAAGAMFLEAIPMS (351–383)	In-house	No
67	IFFKGKWE (212–219)	In-house	No
68	KKLSSWVLLMKYLGNATAIF (257–276)	In-house	No
74	IPPEVKFNKPFV (384–395)	In-house	No
75	IPPEVKF (384–390)	In-house	No
76	QINDYVEKGTQ (180–190)	In-house	No
77	LTIDEKGTEAAG (362–373)	In-house	No
78 = F3	LMIEQNTKSPLFMGKVVNPTQK (397–418)	In-house	No
79	QINDYVEKGTQGK (180–192)	In-house	No
81	MIEQNTKSPL (398–407)	In-house	No
82	LMIEQNTKSPLFMG (397–410)	In-house	No
83	VLTIDEKGTEA (361–371)	In-house	No
84	EQNTKSPLF (400–408)	In-house	No
85	QINDYVEKGTQGKIVD (180–195)	In-house	No
87	SIPPEVKFNKPFVFLMIEQNTKSPLFMGKVVNPTQK (383–418)	In-house	No
88	MFLEAIPMSIPPEVKFNKPFVFLMIEQNTKSPLFMGKVVNPTQK (374–418)	In-house	No
α_1_AT HF	NIQHCKKLSSW (252–262)	Human hemofiltrate library	Yes

### The α_1_AT-derived peptides 42 and 64 inhibit ADP-ribosylation of Gαi by PT in CHO-K1 cells

First, we explored the inhibitory capacity of the published α_1_AT derived peptide VIRIP (Münch et al. 2007) towards PT in a sequential assay analyzing the ADP-ribosylation status of Gαi in PT-treated CHO-K1 cells (Supplementary Fig. 3). In this assay, living cells were first treated with or without PT, cells were lysed, and the cell lysates were treated with recombinant PTS1 ART subunit and biotin-NAD^+^. The non-PT-treated control cells displayed a strong Gαi biotin-ADP-ribose signal, because Gαi in the cell lysate was fully available for ADP-ribosylation by PTS1. In contrast, a weak Gαi biotin-ADP-ribose signal was observed with PT-treated cells, because Gαi had already been ADP-ribosylated in living cells. As a reference and positive control for the inhibition of PT, in this and the following analyses, the full-length plasma-derived α_1_AT, available as a licensed and clinically applied drug (Prolastin®), was used. No significant inhibition of PT was observed with the α_1_AT derived peptide VIRIP, although the full-length plasma-derived α_1_AT strongly inhibited PT. Therefore, the peptide VIRIP, which is located in the C-terminal region of α_1_AT, does not possess anti-PT activity and was not selected for further evaluation.

Next, the α_1_AT-derived peptides F1-5 from the in-house library were screened for their anti-PT activity by analyzing the PT-mediated substrate modification of Gαi in CHO-K1 cells, as performed above (Supplementary Fig. 3). Comparable to VIRIP, the α_1_AT-derived peptides F1–F5 cover different parts of the C-terminal sequence region of the endogenous α_1_AT precursor. The full-length α_1_AT was used as a positive control for the inhibition of PT intoxication. No significant inhibition of PT was observed with the five α_1_AT-derived peptides F1-5, although the full-length plasma-derived α_1_AT strongly inhibited PT (Supplementary Fig. 4). Therefore, the five α_1_AT-derived peptides F1–F5 were not selected for further evaluation.

**Fig. 1:**
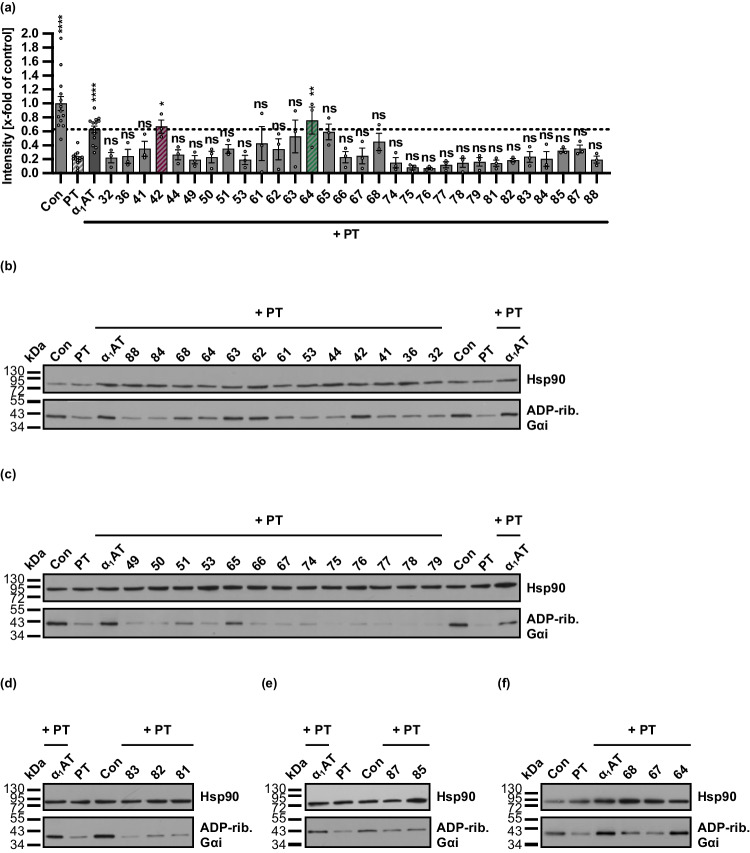
Effect of α_1_AT-derived peptides 32–88 on the ADP-ribosylation status of Gαi in PT-treated CHO-K1 cells. **a–f** PT (10 ng/ml) and 20 µM α_1_AT peptides, 100 µM α_1_AT, or the respective amount of solvent (DMSO) were added directly to CHO-K1 cells in FCS-free medium and incubated for 4 h at 37 °C. For further control cells were left untreated. After the 4 h incubation, cell lysates were generated and Gαi, which had not been ADP-ribosylated during the initial intoxication of living cells with PT, was ADP-ribosylated and biotin-labeled via the subsequent incubation of the cell lysates with recombinant PTS1 and biotin-labeled NAD^+^. Next, the biotin-labeled Gαi was detected via Western blot, while Hsp90 served as a control for equal protein loading. The bar graph (**a**) shows the quantification of Western blot signals from four independent experiments, while (**b**–**f**) show the results of four representative experiments. The intensity values of the bar graph are given as *x*-fold of the untreated control (Con), normalized to Hsp90, mean ± SEM (*n* = 3–14 values from four independent experiments). Significance was tested using one-way ANOVA followed by Dunnett’s multiple comparison test and refers to samples treated with PT only (*p < 0.1, **p < 0.01, ***p < 0.001, ****p < 0.0001, ns not significant)

So far, peptides (VIRIP and F1-F5) covering different regions of the precursor α_1_AT C-terminus, showed no inhibition of PT. Further, 30 in-house peptides (32–88), covering several regions of the full-length sequence of α_1_AT, were selected for analysis of PT inhibition. The same sequential Gαi ADP-ribosylation assay as above (see Supplementary Figs. 3 and 4) was used. A significant inhibition of ADP-ribosylation of Gαi in PT-treated CHO-K1 cells was observed for the two peptides, 42 and 64 (Fig. 1). The other peptides of this region, 61, 62, 63, 65, and 68 but not the peptide 44, also showed a tendency for PT inhibition which, however, was not statistically significant. The remaining peptides, mainly covering the region of the reactive center loop and the C-proximal region of α_1_AT, did not inhibit PT activity.

**Fig. 2:**
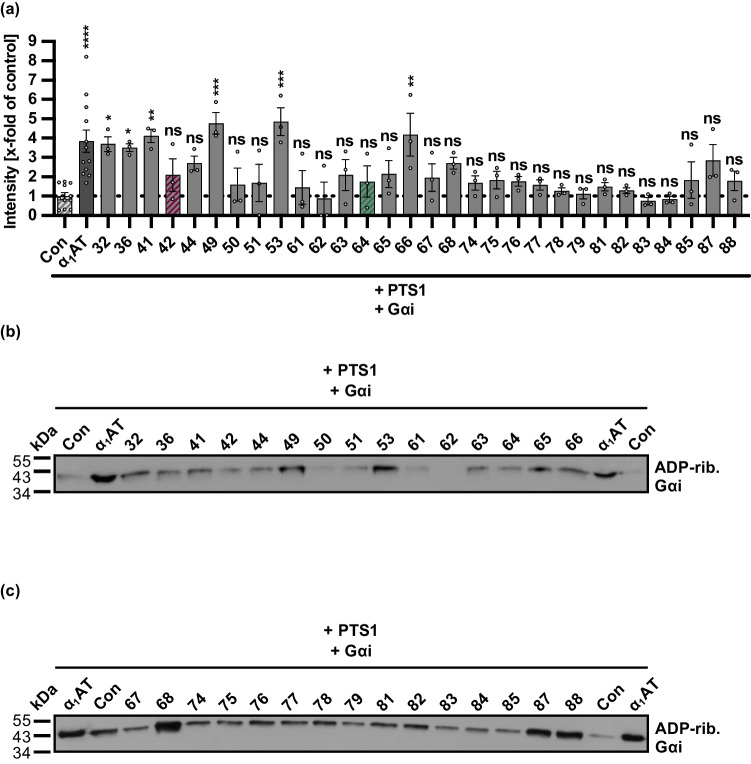
Effect of α_1_AT-derived peptides 32–88 on enzyme activity of PTS1 in vitro.** a–c** PTS1 (84 nM) and 20 µM α_1_AT peptides, 20 µM α_1_AT, or the respective amount of solvent (DMSO) (Con) were preincubated for 15 min at room temperature in buffer and afterwards mixed with Gαi (825 nM) and biotin-NAD^+^ and incubated for a further 40 min at room temperature. During the incubation with recombinant PTS1 and biotin-labeled NAD^+^, Gαi was biotin-ADP-ribosylated. The biotin-labeled Gαi was subsequently detected via Western blot. The bar graph (**a**) shows the quantifications of Western blot signals from five independent experiments, while (**b**, **c**) shows the results of two representative experiments. The intensity values of the bar graph are given as *x*-fold of the untreated control (Con), mean ± SEM (*n* = 3–12 values from five independent experiments). Significance was tested using one-way ANOVA followed by Dunnett’s multiple comparison test and refers to the untreated control (Con) (*p < 0.1, **p < 0.01, ***p < 0.001, ****p < 0.0001, ns not significant)

Additionally, the peptides were tested for their effect on the ART activity of PTS1 in vitro (Fig. 2). Therefore, the peptides were first incubated with recombinant PTS1; subsequently, Gαi and biotin-NAD^+^ were added. Next, the biotin-labeled Gαi was detected via western blot. Most of the tested peptides had no effect on the enzyme activity of PTS1 in vitro. However, for six peptides—peptide 32, 36, 41, 49, 53, and 66—a large increase in the in vitro ART activity of PTS1 was observed. Since these peptides share overlapping amino acid sequences, comparable effects on the PTS1 ART activity are obvious (Supplementary Table 1). Anyhow, the peptides 42 and 64, which showed inhibition of PT during the intoxication of cells, had only minor effects on the ART activity of PTS1 in vitro.

### Endogenous α_1_AT peptide α_1_AT HF from human hemofiltrate inhibited PT activity in CHO-K1 cells

**Fig. 3:**
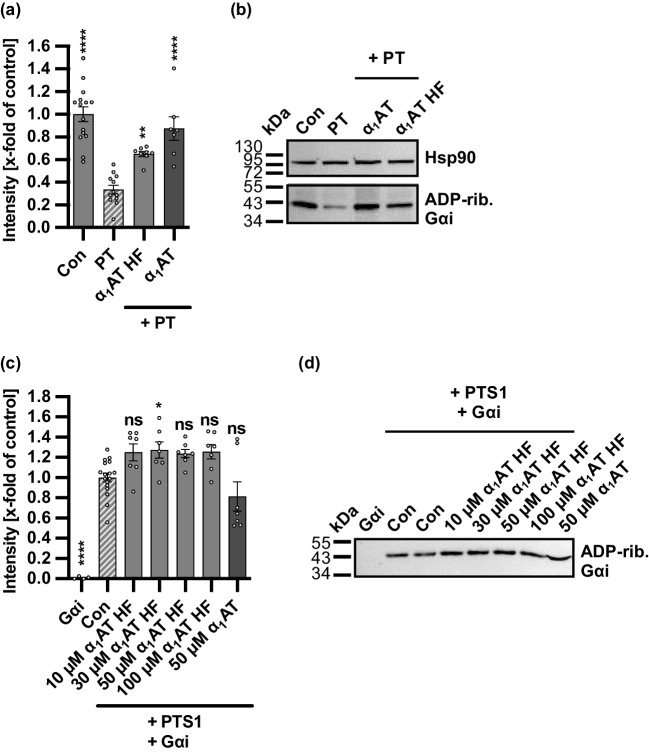
Effect of the endogenous α_1_AT peptide from human hemofiltrate on the ADP-ribosylation status of Gαi in PT-treated CHO-K1 cells and enzyme activity of PTS1 in vitro. **a–b** PT (10 ng/ml) and 100 µM α_1_AT HF peptide, 100 µM α_1_AT, or the respective amount of solvent (H_2_O) were added directly to CHO-K1 cells in FCS-free medium and incubated for 4 h at 37 °C. For further control cells were left untreated. After the 4 h incubation, cell lysates were generated, and Gαi, which had not been ADP-ribosylated during the initial intoxication of living cells with PT, was ADP-ribosylated and biotin-labeled via the subsequent incubation of the cell lysates with recombinant PTS1 and biotin-labeled NAD^+^. Next, the biotin-labeled Gαi was detected via western blot, while Hsp90 served as a control for equal protein loading. The bar graph (**a**) shows the quantification of Western blot signals from four independent experiments, while (**b**) shows the results of a representative experiment. The intensity values of the bar graph are given as *x*-fold of the untreated control (Con), normalized to Hsp90, mean ± SEM (*n* = 7–15 values from four independent experiments). **c–d** PTS1 (125.6 nM) and different concentrations of α_1_AT HF peptide, 50 µM α_1_AT, or the respective amount of solvent (H_2_O) (Con) were mixed with Gαi (825 nM) and biotin-NAD^+^ in buffer and incubated for 40 min at room temperature. As further control Gαi was incubated for 40 min at room temperature with solvent control and biotin-NAD^+^. During the incubation with recombinant PTS1 and biotin-labeled NAD^+^, Gαi was ADP-ribosylated and subsequently detected via Western blot. The bar graph (**c**) shows the quantifications of Western blot signals from four independent experiments, while (**d**) shows the results of a representative experiment. The intensity values of the bar graph are given as *x*-fold of the untreated control (Con), mean ± SEM (*n* = 4–17 values from four independent experiments). Significance was tested using one-way ANOVA followed by Dunnett’s multiple comparison test and refers to samples treated with PT only (**a**, **b**) or untreated control (Con) (**c**, **d**) (*p < 0.1, **p < 0.01, ***p < 0.001, ****p < 0.0001, ns not significant)

Lastly, the effect of the endogenous α_1_AT peptide α_1_AT HF, which was identified from the human hemofiltrate protein/peptide library, was analyzed for its effect on the ADP-ribosylation status of Gαi in PT-treated CHO-K1 cells, as previously described. The endogenous α_1_AT HF peptide covers the same region as the two in-house peptides, 42 and 64, which showed inhibition of PT above (Fig. 1). However, the endogenous α_1_AT HF peptide provided with 11 amino acids is the shortest sequence. A significant inhibition of PT activity was observed with the α_1_AT HF peptide in PT-treated CHO-K1 cells (Fig. 3a, b). Additionally, the effect of the α_1_AT HF peptide on the in vitro enzyme activity of PTS1 was investigated (Fig. 3c, d). Here, for all the tested concentrations of the α_1_AT HF peptide, no inhibitory effect on the enzyme activity of PTS1 was observed.

Since full-length α_1_AT was previously shown to inhibit the binary C2 toxin in a dual mode, by inhibiting C2 binding to target cells and the inhibition of enzyme activity of C2I in vitro (Lietz et al. 2024a), we tested whether the α_1_AT HF peptide is able to inhibit C2 toxin as well (Supplementary Fig. 5). The intoxication of cells with C2 toxin leads to the ADP-ribosylation of G-actin which causes the collapse of the actin cytoskeleton. This in turn causes cell death and thus cell rounding which can easily be monitored using light microscopy. Therefore, HeLa cells were treated with the α_1_AT HF peptide or as a positive control for inhibition of cell rounding with full-length α_1_AT and intoxicated C2 toxin. Here, the α_1_AT HF peptide and the full-length α_1_AT showed inhibition of C2 toxin-mediated cell rounding of HeLa cells.

Consequently, from the two different approaches used to generate peptides with anti-PT, three peptides (42, 64, and α_1_AT HF) were identified that significantly inhibited the intoxication of CHO-K1 cells with PT (Table 1). These three peptides share a common amino acid sequence region, while the α_1_AT HF peptide is the shortest peptide that displayed inhibition of PT and, thus, was considered a lead candidate for further evaluation.

**Fig. 4:**
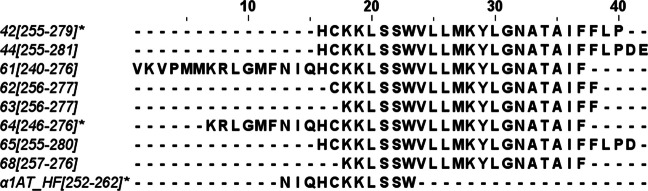
Overview on the nine α_1_AT peptides on the positions 240 to 281 of the α_1_AT precursor (UniProt: P01009-A1AT_HUMAN). Peptide 42, 64, and α_1_AT HF showed significant inhibition of PT, while peptides 61, 62, 63, 65, and 68, showed an attenuated anti-PT activity

Moreover, the region of the three peptides that showed significant inhibition of PT (42, 64, and α_1_AT HF) is also covered by six further peptides, peptides 44, 61, 62, 63, 65, and 68 (Fig. 4). Although not significant, five of those six peptides, peptides 61, 62, 63, 65, and 68, showed a tendency to possess anti-PT activity (Fig. 1).

### Endogenous α_1_AT peptide from human hemofiltrate inhibits the binding of PT to CHO-K1 cells

**Fig. 5:**
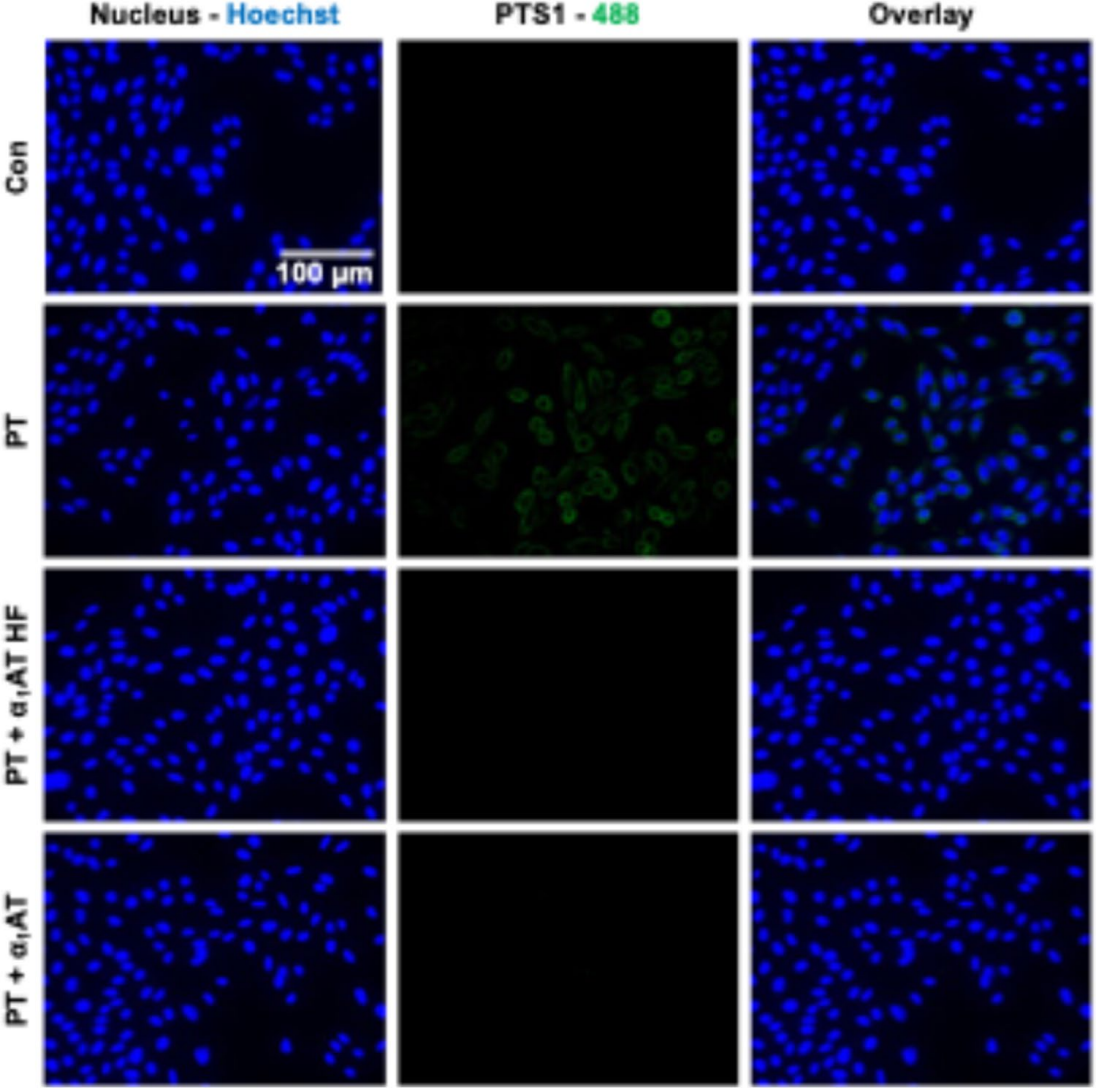
Effect of the endogenous α_1_AT peptide from human hemofiltrate on PT binding to CHO-K1 cells. PT (1 µg/ml), α_1_AT HF, α_1_AT, or the respective amount of solvent (H_2_O) were added to CHO-K1 cells and incubated for 40 min at 4 °C. This enabled PT binding but not the internalization of PT into cells. As further control cells were left untreated. Next, washing, fixation, permeabilization, and quenching were performed. After blocking, the cells were incubated with primary and secondary antibodies to detect PTS1 (green). Nuclei were stained with Hoechst (blue). Representative images are shown from three independent experiments (*n* = 3)

To further investigate the mechanism of inhibition of the α_1_AT peptide from human hemofiltrate towards PT, similarly as before, a fluorescence microscopy-based binding assay was performed (Fig. 5) (Lietz et al. 2024b). Here, for α_1_AT HF and α_1_AT, a reduced signal of PTS1 was observed, meaning the peptide, and the full-length precursor inhibited PT binding to CHO-K1 cells.

### Endogenous α_1_AT peptide from human hemofiltrate has no adverse effects on CHO-K1 cells and zebrafish embryos

**Fig. 6:**
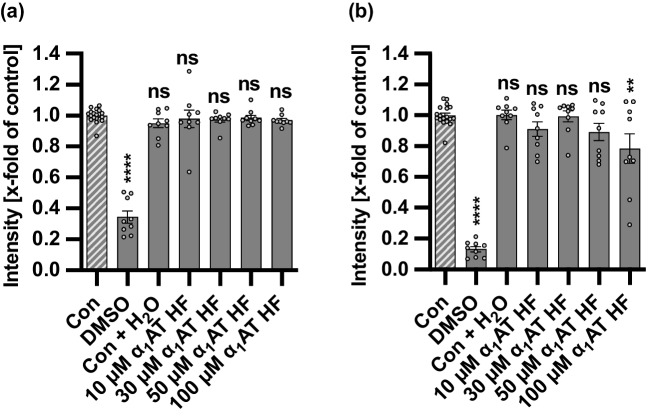
Effect of the endogenous α_1_AT peptide from human hemofiltrate on the cell viability of HeLa cells. **a, b** Different concentrations of α_1_AT HF peptide or the respective amount of solvent (H_2_O) were added directly to HeLa cells in FCS-free medium and incubated for 4 h (**a**) or 24 h (**b**) at 37 °C. As a positive control, cell death was induced by the addition of 20% DMSO. After the respective incubation periods, the MTS reagent was added to the cells and incubated for a further 45 min at 37 °C. The absorbance was measured using a plate reader at 490 nm. The intensity values of the bar graph are given as *x*-fold of the untreated control (Con), mean ± SEM (*n* = 9 values from three independent experiments). Significance was tested using one-way ANOVA followed by Dunnett’s multiple comparison test and refers to the untreated control (Con) (*p < 0.1, **p < 0.01, ***p < 0.001, ****p < 0.0001, ns not significant)

Since the α_1_AT HF peptide identified from the human hemofiltrate library showed inhibition of PT and provided the shortest sequence, it was selected as the lead candidate for further evaluation in an assay analyzing its effect on the cell viability of HeLa cells (Fig. 6). As previously published, we could not identify effects on the cell viability of α_1_AT full-length in the respective assay after 4 and 24 h (Lietz et al. 2024b). Similarly, the α_1_AT HF peptide had no effect on cell viability of HeLa cells after 4 h. Only after 24 h minor effects on cell viability were observed for the highest concentration (100 µM) of the α_1_AT HF peptide.

**Fig. 7:**
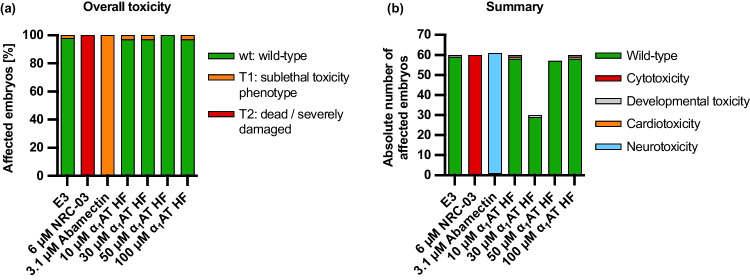
Effect of the endogenous α_1_AT peptide from human hemofiltrate on zebrafish embryos. **a**, **b** α_1_AT HF is not toxic to embryonic zebrafish. Twenty-four hours post-fertilization, dechorionated zebrafish embryos were exposed to DMSO, NRC-03 (cytotoxic control), abamectin (neurotoxic control), or increasing amounts of the indicated peptide for 24 h. Data shown are derived from 60 embryos per group (except for 30 µM α_1_AT HF, which was tested on 30 embryos), sampled in two independent experiments

To further investigate the effects or toxicity of the α_1_AT HF peptide in vivo, we used zebrafish embryos, which combine vertebrate complexity with the ability to perform in vivo assays at high throughput and with large sample sizes (Fig. 7). We exposed embryos for 24 h starting at 24 h post fertilization (hpf), when most organ systems have already developed and are functional. The transparency of the embryos allows for evaluation not only of mortality, but also of sublethal cytotoxicity (necrosis, lysis), developmental toxicity (developmental delay, malformations), or toxicity affecting specific organ systems, in particular cardiotoxicity (heart edema, reduced circulation) and neurotoxicity (reduced touch escape response, Supplementary Fig. 6, Supplementary Table 2). The different tested concentrations of the α_1_AT HF peptide were not toxic in zebrafish embryos (Fig. 7).

### Most of the amino acids of endogenous α_1_AT peptide α_1_AT HF are crucial for the inhibition of PT

An alanine exchange scan was performed to further explore the inhibitory effect of the endogenous α_1_AT HF peptide on PT and to potentially identify the amino acids relevant to the inhibition of PT. Therefore, the amino acids of each position of the α_1_AT HF peptide were exchanged for alanine, resulting in eleven peptides (α_1_AT HF P1-11, see Table 2).

**Table 2:** Peptides resulting from the alanine scan of the α_1_AT HF peptide (NIQHCKKLSSW) that were tested for their inhibition of PT. The peptides are listed with their name, the sequence, and their anti-PT activity

Peptide name	Sequence	Anti-PT activity
α_1_AT HF P1	AIQHCKKLSSW	No
α_1_AT HF P2	NAQHCKKLSSW	No
α_1_AT HF P3	NIAHCKKLSSW	No
α_1_AT HF P4	NIQACKKLSSW	No
α_1_AT HF P5	NIQHAKKLSSW	No
α_1_AT HF P6	NIQHCAKLSSW	No
α_1_AT HF P7	NIQHCKALSSW	No
α_1_AT HF P8	NIQHCKKASSW	Yes
α_1_AT HF P9	NIQHCKKLASW	No
α_1_AT HF P10	NIQHCKKLSAW	No
α_1_AT HF P11	NIQHCKKLSSA	No

**Fig. 8:**
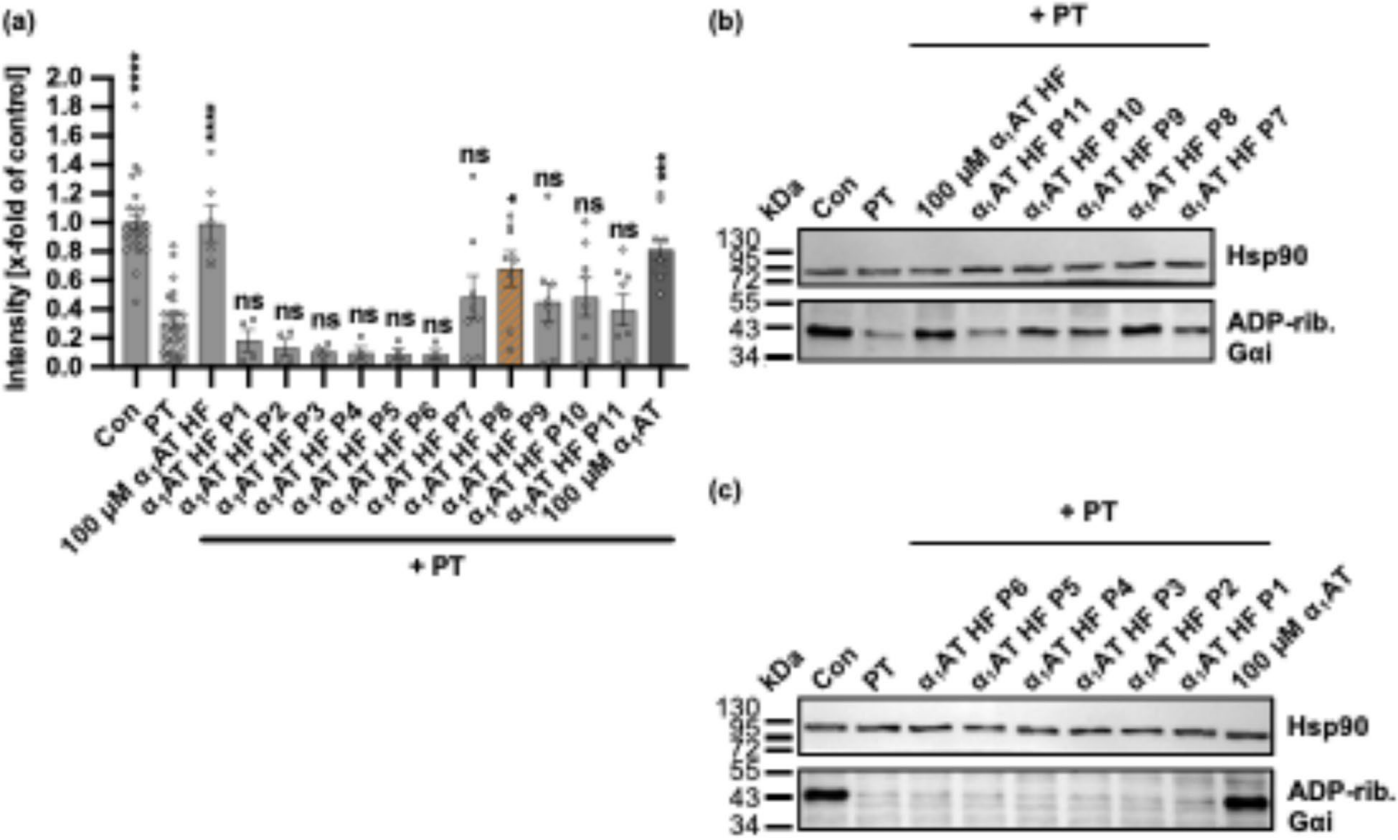
Effect of the substitution of amino acids with alanine of endogenous α_1_AT peptide from human hemofiltrate on ADP-ribosylation of Gαi in PT-treated CHO-K1 cells. **a–c** PT (10 ng/ml) and 100 µM α_1_AT HF peptide, 200 µM α_1_AT HF peptide P1-11, 100 µM α_1_AT, or the respective amount of solvent (H_2_O) were added directly to CHO-K1 cells in FCS-free medium and incubated for 4 h at 37 °C. For further control cells were left untreated. After the 4-h incubation, cell lysates were generated, and Gαi, which had not been ADP-ribosylated during the initial intoxication of living cells with PT, was ADP-ribosylated and biotin-labeled via the subsequent incubation of the cell lysates with recombinant PTS1 and biotin-labeled NAD^+^. Next, the biotin-labeled Gαi was detected via western blot, while Hsp90 served as a control for equal protein loading. The bar graph (**a**) shows the quantification of Western blot signals from three independent experiments, while (**b**, **c**) show the results of a representative experiment. The intensity values of the bar graph are given as *x*-fold of the untreated control (Con), normalized to Hsp90, mean ± SEM (*n* = 4–24 values from six independent experiments). Significance was tested using one-way ANOVA followed by Dunnett’s multiple comparison test and refers to samples treated with PT only (*p < 0.1, **p < 0.01, ***p < 0.001, ****p < 0.0001, ns not significant)

These peptides were tested using the same assay as employed before, analyzing the ADP-ribosylation status of Gαi in PT-treated CHO-K1 cells (Fig. 8, Supplementary Fig. 7). As a positive control for inhibition of PT, the full-length α_1_AT and the α_1_AT HF peptide were used. All peptides, α_1_AT HF P1–11, except the peptide α_1_AT HF P8 (NIQHCKKASSW), did not inhibit PT.

## Discussion

Pertussis is a life-threatening, highly infectious respiratory disease caused by the bacterium *B. pertussis* via its exotoxin PT. PT is associated with characteristic symptoms of pertussis and bears an ADP-ribosyltransferase activity. Currently, and despite the vaccination, case numbers of pertussis are on the rise again, also in Western countries (European Centre for Disease Prevention and Control 2024). Facing these increasing case numbers and the lack of targeted treatment strategies, novel strategies for the disease management in the clinics are urgently required.

### Finding the anti-PT region of α_1_AT

To develop novel pharmacological options against PT, our research focus is on the discovery of potent and specific inhibitors of PT in human body fluids. Using this approach, we recently identified endogenous α_1_AT as an inhibitor for PT through the screening of a human hemofiltrate protein/peptide library (Lietz et al. 2024b). We could further show that the mechanism of PT inhibition relied on the inhibition of PT binding to target cells and is most likely mediated via direct interaction of the pentameric B-subunit of PT with α_1_AT (Lietz et al. 2024b). Moreover, other bacterial toxins were also inhibited by endogenous α_1_AT, including C2 toxin, DT, and an anthrax fusion toxin (Lietz et al. 2024a). As especially the B-components of C2 toxin and anthrax toxin, C2IIa and PA63, respectively, were inhibited by α_1_AT, the results suggest a common interaction site of the B-subunits of bacterial toxins with α_1_AT. Based on our findings, we were primarily interested in the amino acid sequence within α_1_AT that possesses the anti-PT activity. Therefore, we evaluated the anti-PT activity of α_1_AT fragments and screened them experimentally by analyzing the ADP-ribosylation status of Gαi in PT-treated CHO-K1 cells. For the α_1_AT peptide design, two different approaches were employed, resulting in a total of 36 screened peptides derived from α_1_AT.

Based on the screening results with these peptides, we were able to narrow down the PT inhibitory region to 42 amino acids, which are the amino acids at positions 240 to 281 of the α_1_AT precursor. Interestingly, no specific function can be attributed to this region, although there is a cysteine residue (aa position 256) and a glycosylation site (aa position 271) (UniProt: P01009-A1AT_HUMAN). The respective inhibitory region was covered by a total of 9 different peptides with variation in length. For the peptides 42 and 64, as well as for the endogenous α_1_AT HF peptide (found in hemofiltrate), significantly less ADP-ribosylated Gαi was detected in PT-treated CHO-K1 cells, indicating their PT-inhibiting activities. The amino acids from position 240 to 281 are relatively exposed to the surface of α_1_AT in its folded conformation and additionally show proximity to the reactive center loop of α_1_AT, as evident from multiple experimentally determined crystal structures, e.g., PDB-1PSI (Elliott et al. 1996).

However, none of the other synthetic α_1_AT fragments inhibited PT in the screening assay. Also, peptides covering the region of the reactive center loop of α_1_AT at the C-terminal region with the reactive methionine residue at position 358 (equals position 382 from sequence UniProt: P01009-A1AT_HUMAN) (Loebermann et al. 1984; O’Brien et al. 2022), showed no inhibition of PT. This also includes the known peptide sequence termed VIRIP (α_1_AT precursor 377–396), an α_1_AT peptide derived from the C-terminal part of α_1_AT that inhibits HIV-1 by interacting with the gp41 fusion peptide (Münch et al. 2007), which did not inhibit PT. This distinguishes the interaction site of α_1_AT responsible for HIV-1 inhibition from the interaction site responsible for bacterial toxin inhibition.

The reactive center loop of α_1_AT displays a central structure for the execution of α_1_AT’s function as a protease inhibitor. In the native conformation of α_1_AT, the reactive center loop is exposed as a pseudo-substrate for its target protease (Kelly-Robinson et al. 2021). After the binding of the target protease to the reactive center loop, α_1_AT undergoes a major conformational shift which results in the covalent binding and integration of the target protease into the β-sheet of α_1_AT (Kelly-Robinson et al. 2021). Through this reaction, the target protease is trapped and inactivated. Since peptides covering the region of the reactive center loop did not inhibit PT, PT might not serve as a target structure for the reactive center loop of α_1_AT. This hypothesis is further supported by earlier data which suggest that the protease inhibition activity of α_1_AT is not relevant for the inhibition of PT. In silico modeling of the α_1_AT-PT interaction showed that none of the amino acids of the reactive center loop of α_1_AT mediated the interaction with PT (Lietz et al. 2024b). In addition, another member of the serpin family with protease activity, antithrombin, was not able to inhibit PT (Lietz et al. 2024b).

### Ala-scanning and the anti-PT activity

Since the endogenous α_1_AT HF peptide was the shortest peptide (11 amino acid residues) with anti-PT activity, we considered it as a lead candidate for further evaluation. Fluorescence microscopy-based binding experiments showed that the α_1_AT HF peptide inhibited PT binding to CHO-K1 cells. Moreover, its effect on cell viability and in vivo on zebrafish embryos was tested. The results showed no toxic effects of the α_1_AT HF peptide, rendering it promising for further improvement. An alanine scan was performed to identify which amino acids of the α_1_AT HF peptide are crucial for the anti-PT activity. The replacement of every amino acid residue with alanine generated 11 further derivatives (α_1_AT HF P1-11) that were tested for their inhibition of PT. This resulted in the loss of anti-PT activity in the screening assay, except for the peptide modified at position 8, α_1_AT HF P8, which inhibited PT-mediated ADP-ribosylation of Gαi. This indicates that almost all amino acid residues of the α_1_AT HF peptide, except the leucine at position 8, are relevant for inhibition of PT, since the exchange of these amino acids resulted in the loss of anti-PT activity. The leucine at position 8 seems not to be relevant for inhibition of PT, since the peptide α_1_AT HF P8 significantly inhibited PT-mediated ADP-ribosylation of Gαi. Moreover, the α_1_AT HF peptide bears a cysteine residue in the second position, which is relevant for PT inhibition, maybe due to its reactivity and the possibility of forming disulfide bridges. Further replacements of Leu8 for other residues should be investigated to obtain variants with higher anti-PT activity.

However, the results of the biological evaluation of those peptides with sequences covering partially or completely the core region 252–262 suggest that other residues may influence the interaction with the molecular target (Fig. 5). For example, in contrast to our finding by Ala-scanning, peptide 42 (255–279) lacks the segment NIQ, retained the anti-PT activity, suggesting that at least part of the very hydrophobic region VLLMKYLGNATAIFFLP may also be relevant for the biological activity. On the other hand, the non-active peptides 65 (255–280) and 44 (255–281) have the same N-terminus as 42, being only one (D) and two acidic residues (DE) longer than 42 at the C-terminus, respectively. The reduced activity of peptides 65 and 44 suggests that negatively charged residues could somewhat disfavor the interaction with the molecular target and, therefore, the anti-PT activity.

Compared to the active peptide 42, the non-active peptides 62, 63, 64, and 68 lack the relevant N-terminal residues H or HC and the C-terminal residues LP or FLP. On the other hand, the active peptide 64 contains the active core region NIQHCKKLSSW and is not affected by the lack of the C-terminal residues FLP, which suggests they are not critical for the activity and/or the N-terminal elongation (KRLGMF) favors the interaction with the target or at least does not interfere with it. In contrast, the non-active peptide 61, which contains the core region NIQHCKKLSSW and has the same C-terminus as the active peptide 64, has a longer N-terminus with the additional region VKVPMM, which seems to interfere with the activity.

Although the presence or absence of several N- or C-terminal residues provides some clues on their influence on the activity, additional structure–activity studies will be required to identify these residues beyond the region 252–262. Also, further experiments, e.g., using scrambled or truncated variants of NIQHCKKLSSW, will show whether the sequence of the amino acids is relevant for the anti-PT activity and/or can be further shortened.

In conclusion, the region of α_1_AT that possesses anti-PT activity is located rather in the middle of the endogenous protein, which is in spatial proximity to the reactive center loop (α_1_AT precursor 368–392) which is not involved in the anti-PT activity. The biological evaluation of the region was covered by testing nine peptides, with three peptides showing significant inhibition, 42, 64, and α_1_AT HF. The lead candidate that showed inhibition of PT with the shortest sequence, α_1_AT HF, was further evaluated. The α_1_AT HF peptide does not inhibit the enzyme activity of PTS1 in vitro and has no cytotoxic effect on the cell viability of HeLa cells. Moreover, the peptide showed no toxic effects in zebrafish embryos. Almost all amino acids of the peptide are relevant for the inhibition of PT. This renders the peptide a novel pharmacological inhibitor for the *B. pertussis* toxin and an interesting candidate for further evaluation in vivo to combat pertussis, for example, in the infant mouse model for pertussis.

## Experimental procedures

### Compounds and reagents

The *B. pertussis* toxin (PT) used for the experiments was purchased from Sigma-Aldrich, Merck. Moreover, the recombinantly produced proteins Gαi and PTS1 were expressed and purified as described earlier (Ashok et al. 2020). The commercially available drug Prolastin® purchased from Grifols served as a source for α_1_AT.

### Identification of α_1_AT peptides from human hemofiltrate

A search to find α_1_AT fragments in hemofiltrate from the sequencing data of several projects within the CRC1279 (https://www.uni-ulm.de/en/med/crc-1279/) yielded 92 peptide sequences. A multiple sequence alignment was constructed with Clustal Omega (https://www.ebi.ac.uk/jdispatcher/msa/clustalo, (Madeira et al. 2024)) using the 92 sequences from hemofiltrate and in-house peptide sequences HCKKLSSWVLLMKYLGNATAIFFLP and KRLGMFNIQHCKKLSSWVLLMKYLGNATAIF since they showed inhibitory activity on PT. Jalview version 2.11.4.1 (https://www.jalview.org/, (Waterhouse et al. 2009)) was used to visualize the multiple sequence alignment.

### Synthesis of α_1_AT peptides

NIQHCKKLSSW was synthesized in the Core Facility Functional Peptidomics (Ulm University) by standard Fmoc solid-phase peptide synthesis using a Liberty Blue microwave synthesizer (CEM, USA) as previously described (Harms et al. 2021).

### Cell lines

For the cultivation of cell lines all materials were purchased from Gibco (Thermo Fisher Scientific, Waltham, MA, USA), unless indicated differently. The experimentally used cell lines CHO-K1 (Chinese hamster ovary cells strain K1; DSMZ) and HeLa cells (human cervical carcinoma cells; DSMZ, Braunschweig, Germany) were cultivated under humidified conditions at 37 °C with 5% CO_2_. For the cultivation of CHO-K1 cells Dulbecco’s modified Eagle’s medium and HAM’s F12 (1:1), supplemented with 5% FCS, 1 mM sodium pyruvate, 0.05 mM nonessential amino acids, and 100 U/ml penicillin and 100 g/ml streptomycin were used, while HeLa cells were cultivated in minimum essential medium (MEM), supplemented with 10% FCS, 1 mM sodium pyruvate, 0.1 mM non-essential amino acids, and 100 U/ml penicillin and 100 g/ml streptomycin. During the cultivation of the cells, they were trypsinized and reseeded every 2 to 3 days with a maximum of 25 times. For the performed intoxication experiments, the cells were seeded in culture dishes 1 or 2 days before and treated in FCS-free media with toxins and the respective compounds.

### Cell viability assay

To examine the effect of a peptide on cell viability, HeLa cells were seeded in 96-well plates one day before treatment with the peptide, water (solvent control), and 20% dimethylsulfoxide as a positive control for cell death in FCS-free medium for 4 or 24 h. After that the cell viability was determined using the CellTiter 96 Aqueous One Solution cell proliferation assay (MTS assay, Promega), which was added to the cells and incubated for 45 min at 37 °C. Finally, the absorbance was measured using a plate reader at 490 nm.

### Sequential ADP-ribosylation of Gαi in PT-treated cells

To examine the ADP-ribosylation status of Gαi in the presence of different peptides and PT a sequential ADP-ribosylation assay was carried out. The sequential ADP-ribosylation assay is a Western blot-based method. Therefore, CHO-K1 cells were seeded in 24-well plates one day before treatment with the peptides, α_1_AT, water/PBS/DMSO (solvent controls), and PT in FCS-free medium. The volume of the solvent control was equal to the highest volume of peptides added during the assay. After 4 h of treatment with the compounds at 37 °C, cells were washed, and the cell lysates were collected in ADP-ribosylation buffer (0.1 mM Tris–HCl (pH 7.6), 20 mM DTT, 0.1 mM ATP, and protease inhibitor complete (Roche)). For the execution of the sequential in vitro ADP-ribosylation of Gαi, which was not modified due to the previous intoxication of cells with PT, recombinant PTS1 (95.7 nM) was used. The reaction of the ADP-ribosylation was labeled using biotin-NAD^+^ (1 mM; R&D Systems) during the 40 min incubation at room temperature. Subsequently, the samples were denatured for gel electrophoresis, and Western blotting was performed. The biotin-labeled and thus sequentially ADP-ribosylated Gαi was detected using streptavidin-peroxidase (Strep-POD, Sigma-Aldrich, Merck). Hsp90 (primary antibody from Santa Cruz Biotechnology) served as a loading control. Finally, the quantification of signals was performed using the ImageJ software v.1.52.a (National Institutes of Health, Bethesda (NIH)). Here, the Hsp90 signal intensity was used to normalize Gαi signal intensity.

### Enzyme activity assay of PTS1 with purified Gαi

The in vitro enzyme activity of PTS1 was assessed in the presence of different peptides, using recombinant Gαi. Therefore, the different peptides, water/DMSO (solvent controls), and recombinant PTS1 (84 nM) were added to 50 mM sodium phosphate buffer (pH 7.0) and incubated for 15 min at room temperature. The volume of the solvent control was equal to the highest volume of peptides added during the assay. After that, recombinant Gαi (825 nM) and biotin-labeled NAD^+^ (1 mM; R&D Systems) were added to mark the ADP-ribosylation of Gαi during the 40 min incubation at room temperature. For the α_1_AT from hemofiltrate, no preincubation with PTS1 was performed, and all components were directly mixed and incubated for 40 min. After that, the samples were subjected to gel electrophoresis and Western blotting. The ADP-ribosylated Gαi by PTS1 was detected using streptavidin-peroxidase (Strep-POD, Sigma-Aldrich, Merck), and the signal quantification was performed using the ImageJ software v.1.52.a (NIH). To control the equal loading of samples, Ponceau-S staining was performed.

### Gel electrophoresis and western blotting

For the sequential ADP-ribosylation of Gαi in PT-treated cells and enzyme activity assay of PTS1 with purified Gαi, gel electrophoresis and Western blotting were conducted. After the samples were prepared, Laemmli buffer (0.3 M Tris–HCl, 10% SDS, 37.5% glycerol, 0.4 mM bromophenol blue, 100 mM DTT) was added, and the samples were heat-denatured at 95 °C for 10 min. Next, the samples were subjected to gel electrophoresis. Here, the proteins were separated using 12.5% acrylamide gels, while the transfer of proteins from the gels onto nitrocellulose membranes was conducted using semi-dry western blotting. The blotting procedure was controlled by Ponceau-S staining (AppliChem GmbH) of the membranes. Next, the membranes were blocked in 5% skim milk powder solved in PBS-T (PBS containing 0.1% Tween 20) for at least 30 min at room temperature. After the blocking step, the membranes were washed with PBS-T. Then, the membranes were incubated with primary antibodies and washed again before the addition of the secondary antibodies. Before the detection of the signals the membranes were washed again. The signals were detected using Pierce ECL Western blotting substrate (Thermo Fisher Scientific) and X-ray films (AGFA HealthCare) or the iBright 1500 system (Thermo Fisher Scientific).

### Cell morphology assay

For the analysis of cell morphological changes, HeLa cells were seeded in 96-well plates 1 or 2 days before treatment with the α_1_AT HF peptide, α_1_AT, water (solvent controls), and C2 toxin in FCS-free medium. The volume of the solvent control was equal to the highest volume of peptides added during the assay. After the treatment of the cells, the cell morphological changes were documented using light microscopy hourly over a total period of 7 h, using a Leica DMi1 microscope connected to a Leica MC170 HD camera (both Leica Microsystems GmbH, Wetzlar, Germany). Rounded, intoxicated and non-rounded, non-intoxicated cells were counted using the online software Neuralab (https://neuralab.de).

### Fluorescence microscopy experiments

Fluorescence microscopy experiments were performed to investigate the binding of PT to CHO-K1 cells. Therefore, cells were seeded in 18-well µm slides (ibidi GmbH) and treated with α_1_AT HF, α_1_AT, or water (solvent control) and PT in FCS-free medium for 40 min at 4 °C. The volume of the solvent control was equal to the highest volume of peptides added during the assay. Subsequently, cells were washed with PBS, fixed with 4% paraformaldehyde for 20 min, permeabilized using 0.4% (v/v) Triton X-100 in PBS for 5 min, and quenching was performed for 2 min in glycine (100 mM in PBS). After that blocking was performed for 1 h at 37 °C in PBS-T (PBS containing 0.1% Tween 20) containing 10% normal goat serum (Jackson ImmunoResearch) and 1% bovine serum albumin. For the detection of PT, the cells were incubated with the primary antibody for PTS1 (anti-PTS1, clone 63.1G9, sc-57639, Santa Cruz). The primary antibody was detected via fluorescently labeled secondary antibody, and cell nuclei were stained for 5 min using Hoechst 33,342 (1:10,000, Thermo Fisher Scientific). After staining, microscopy was performed with the BZ-X810 Keyence fluorescence microscope (Keyence Deutschland GmbH, Neu-Isenburg) and BZ-X800Viewer v1.3.0.

### Zebrafish experiments

For in vivo studies, wild-type zebrafish embryos (*Danio rerio*) were dechorionated at 24 h post fertilization (hpf) using digestion with 1 mg/ml pronase (Sigma) in E3 medium (83 μM NaCl, 2.8 μM KCl, 5.5 μM 202 CaCl2, 5.5 μM MgSO4). In a 96-well plate, three embryos per well were exposed for 24 h to 100 µL of E3 containing α_1_AT HF peptide at the concentrations indicated in the figures. Two independent assays were performed, each with 10 × 3 embryos. The peptide was dissolved in water and diluted in E3; therefore E3 was used as a negative control. As a positive control for acute toxicity/cytotoxicity the pleurocidin antimicrobial peptide NRC-03 (GRRKRKWLRRIGKGVKIIGGAALDHL-NH2) was used at a concentration of 3 µM as described (Morash et al. 2011). Abamectin at a concentration of 3.125 µM was used as a positive control for neurotoxicity (Raftery et al. 2014). At 48 hpf (after 24 h of incubation), embryos were scored in a stereomicroscope for signs of cytotoxicity (lysis and/or necrosis), developmental toxicity (delay and/or malformations), or cardiotoxicity (heart edema and/or reduced or absent circulation). Each embryo was also touched with a needle, and reduced or absent touch response (escape movements) was evaluated as evidence for neurotoxicity if and only if no signs of acute toxicity were present in the same embryo. Embryos were categorized within each of these toxicity categories into several classes of severity according to Supplementary Table 1. Fisher’s exact test was used to calculate whether the distribution of embryos into toxicity classes differed significantly between the E3 negative control and the test substances.

### Reproducibility of experiments and statistics

All performed experiments were conducted independently from each other at least three times. The number of replicates (*n*) for experiments or tested conditions is given in the figure legends, while representative results are shown in the figures. If not stated otherwise in the figure legends, the statistical analysis performed was a one-way ANOVA in combination with Dunnett’s multiple comparison test using GraphPad Prism Version 9 (GraphPad Software Inc., San Diego, CA, USA). The obtained *p* values are depicted as follows: ns = not significant; *p* > 0.05, *p < 0.05, **p < 0.01, ***p < 0.001, ****p < 0.0001.

## Supplementary Information

Below is the link to the electronic supplementary material.

ESM 1 (DOCX 3.38 MB)

## Data Availability

All source data for this work (or generated in this study) are available upon reasonable request.

## Abbreviations

α_1_AT: α_1_-Antitrypsin
ART: ADP-ribosyltransferase
Gαi: α-Subunit of inhibitory G proteins
AC: Adenylate cyclase
B.anthracis: *Bacillus anthracis*
*B. pertussis*: *Bordetella pertussis*
BAL: Bronchoalveolar lavage
CHO-K1 cells: Chinese hamster ovary cells strain K1
*C. botulinum*: *Clostridium botulinum*
*C. diphtheriae*: *Corynebacterium diphtheriae*
ER: Endoplasmic reticulum
ERAD: ER-associated degradation
ECDC: European Centre for Disease Prevention and Control
GPCRs: G protein-coupled receptors
hpf: Hours post fertilization
PT: Pertussis toxin
VIRIP: Virus-inhibitory peptide
WB: Western blot
