# Supplementary material for: An endogenous human peptide derived from α1-antitrypsin as a novel pharmacological inhibitor of Bordetella pertussis toxin
Source: Naunyn Schmiedebergs Arch Pharmacol. 2025 Nov 17;399(4):5987–6002. doi: 10.1007/s00210-025-04744-1 (PMC13046643; doi:10.1007/s00210-025-04744-1)
Supplement: Supplementary file 1 — (DOCX 3.38 MB) [file 210_2025_4744_MOESM1_ESM.docx]

**Supplementary Figures – An endogenous human peptide derived from α_1_-antitrypsin as novel pharmacological inhibitor of *Bordetella pertussis* toxin**

**
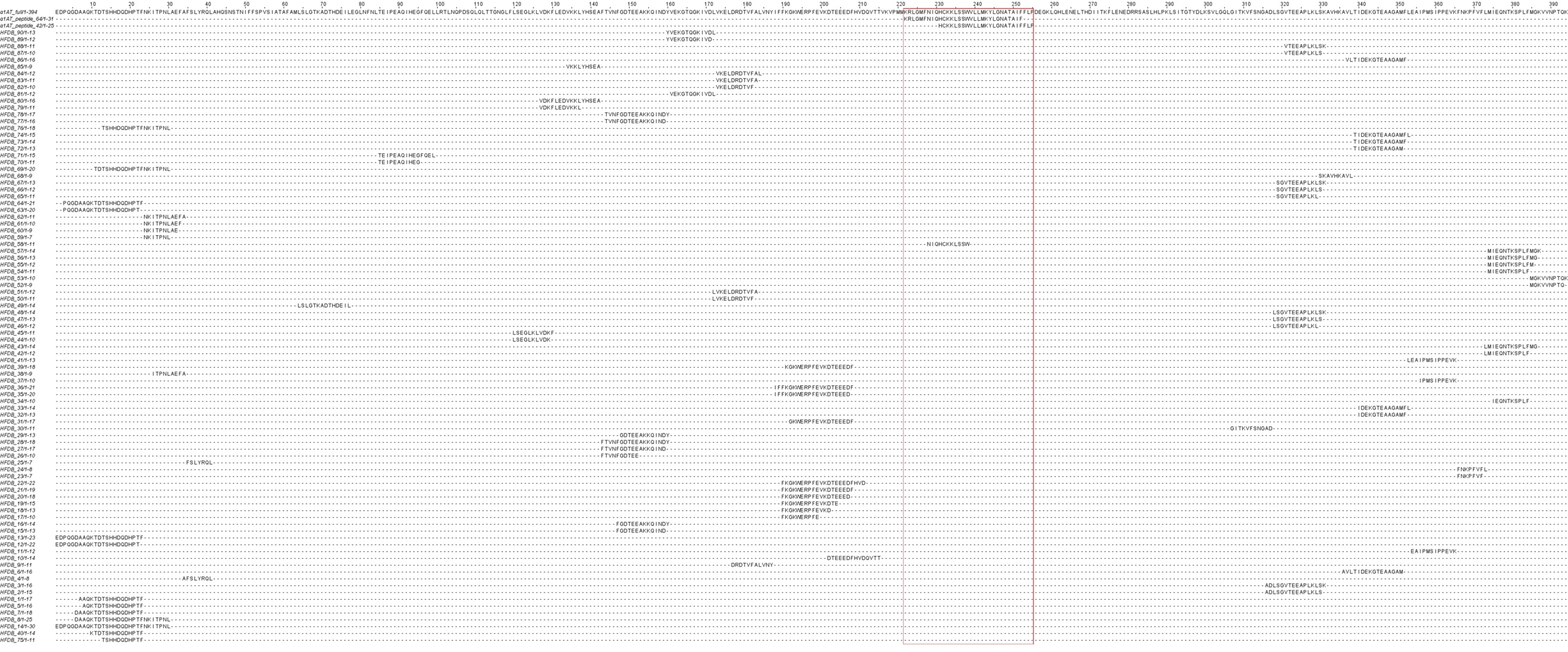
**

**Supplementary Figure 1. Multiple sequence alignment of in-house peptides derived from α_1_AT with α_1_AT peptides found in the human hemofiltrate library.** Amino acid sequence alignment of the two in-house peptides derived from α_1_AT, peptide 42 and 64, that showed inhibition of PT with α_1_AT derived peptides found in the human hemofiltrate library.

**
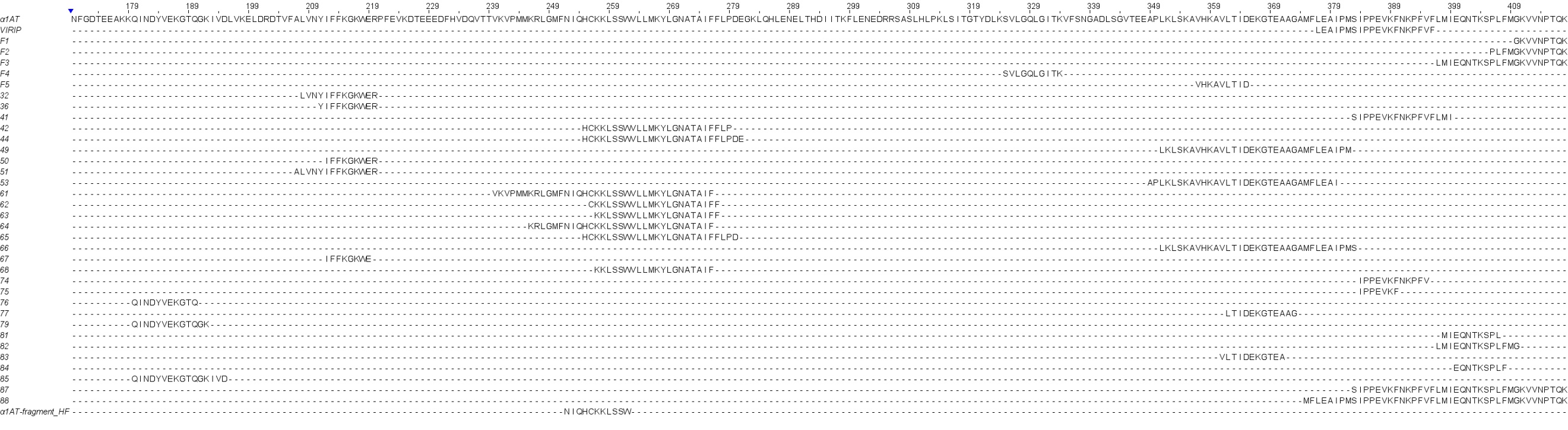
**

**Supplementary Figure 2. Overview on all α_1_AT peptides.** Amino acid sequence alignment of tested peptides derived from α_1_AT with the full-length endogenous α_1_AT (UniProt: P01009- A1AT_HUMAN). The two α_1_AT-derived peptides 42 and 64 showed inhibition of PT.

**Supplementary Figure 3: Effect of VIRIP on the ADP-ribosylation status of Gαi in PT-treated CHO-K1 cells. (a-c)** PT (10 ng/ml) and different concentrations of VIRIP, 100 µM α_1_AT or the respective amount of solvent (PBS) were added directly to CHO-K1 cells in FCS-free medium and incubated for 4 h at 37 °C. For further control cells were left untreated. After the 4 h incubation, cell lysates were generated and Gαi, which had not been ADP-ribosylated during the initial intoxication living cells with PT, was ADP-ribosylated and biotin-labeled via the subsequent incubation of the cell lysates with recombinant PTS1 and biotin-labeled NAD^+^. Next, the biotin-labeled Gαi was detected via Western blot, while Hsp90 served as a control for equal protein loading. The bar graph (a) shows the quantification of Western blot signals from six independent experiments, while (b, c) show the results of two representative experiments. The intensity values of the bar graph are given as x-fold of the untreated control (Con), normalized to Hsp90 or Ponceau-S staining, mean ± SEM (n = 3-21 values from six independent experiments). Significance was tested using one-way ANOVA followed by Dunnett’s multiple comparison test and refers to samples treated with PT only (*p < 0.1, **p < 0.01, ***p < 0.001, ****p < 0.0001, ns not significant).

**Supplementary Figure 4: Effect of the peptides F1-5 derived from α_1_AT on the ADP-ribosylation status of Gαi in PT-treated CHO-K1 cells. (a-b)** PT (10 ng/ml) and 100 µM α_1_AT peptides F1-F5, 100 µM α_1_AT or the respective amount of solvent (H_2_O) were added directly to CHO-K1 cells in FCS-free medium and incubated for 4 h at 37 °C. For further control cells were left untreated. After the 4 h incubation, cell lysates were generated and Gαi, which had not been ADP-ribosylated during the initial intoxication of living cells with PT, was ADP-ribosylated and biotin-labeled via the subsequent incubation of the cell lysates with recombinant PTS1 and biotin-labeled NAD^+^. Next, the biotin-labeled Gαi was detected via Western blot, while Hsp90 served as a control for equal protein loading. The bar graph (a) shows the quantification of Western blot signals from four independent experiments, while (b) shows the results of a representative experiment. The intensity values of the bar graph are given as x-fold of the untreated control (Con), normalized to Hsp90, mean ± SEM (n = 8-16 values from four independent experiments). Significance was tested using one-way ANOVA followed by Dunnett’s multiple comparison test and refers to samples treated with PT only (*p < 0.1, **p < 0.01, ***p < 0.001, ****p < 0.0001, ns not significant).

**Supplementary Table 1: Peptides that increased ART activity of PTS1 *in vitro* have overlapping sequences.** The overlapping amino acids are highlighted in yellow, green, blue, and red.

| **Peptide name** | **Sequence (positions in α_1_AT precursor sequence 1-418, UniProt ID P01009)** | **Sequence overlap** |
| --- | --- | --- |
| **32** | LVNYIFFKGKWER (208-220) | Yes, with 36 |
| **36** | YIFFKGKWER (211-220) | Yes, with 32 |
| **49** | LKLSKAVHKAVLTIDEKGTEAAGAMFLEAIPM (351-382) | Yes, with 53 and 66 |
| **53** | APLKLSKAVHKAVLTIDEKGTEAAGAMFLEAI (349-380) | Yes, with 49 and 66 |
| **66** | LKLSKAVHKAVLTIDEKGTEAAGAMFLEAIPMS (351-383) | Yes, with 49, 53, and 41 |
| **41** | SIPPEVKFNKPFVFLMI (383-399) | Yes, with 66 |

**Supplementary Figure 5. Effect of endogenous α_1_AT peptide from human hemofiltrate on C2 mediated cell rounding of HeLa cells. (a-c)** C2 (C2I: 100 ng/ml + C2IIa: 200 ng/ml), 100 µM α_1_AT, 100 µM α_1_AT HF or the respective amount of its solvent (H_2_O) were added together in FCS-free medium to HeLa cells. The cells were incubated with the substances for 7 h at 37 °C, and pictures were taken every hour. Rounded cells are given as percent of the total cell count, mean +/- SEM (n = 9 values from three independent experiments, each performed with triplicates), for the time course of the whole experiment (a) or for the endpoint after 7 h (b). Representative images are shown after 7 hours (c). Significance was tested using one-way ANOVA followed by Dunnett’s multiple comparison test and refers to C2 toxin treated controls (C2) (* p < 0.1, ** p < 0.01, *** p < 0.001, **** p < 0.0001, ns not significant).

**Supplementary Figure 6: Effect of the endogenous α_1_AT peptide from human hemofiltrate on zebrafish embryos. (a-e)** α_1_AT HF is not toxic to embryonic zebrafish. Twenty-four hours post-fertilization, dechorionated zebrafish embryos were exposed to DMSO, NRC-03 (cytotoxic control), abamectin (neurotoxic control), or increasing amounts of the indicated peptide for 24 hours. Toxicity divided into (a) cytotoxicity (lysis), (b) cytotoxicity (necrosis), (c) developmental toxicity, (d) cardiotoxicity, and (e) neurotoxicity is shown. Data shown are derived from 60 embryos per group, sampled in two independent experiments.

**Supplementary Table 2. Scoring criteria for zebrafish experiments.** Classes of phenotypic severity are given for cytotoxicity, developmental toxicity, cardiotoxicity, neurotoxicity, and overall toxicity.

| **Cytotoxicity** | |
| --- | --- |
| L1: | few lysed cells floating in medium; embryos look like wt |
| L2: | lysed cells in medium; embryos show some visible tissue damage |
| L3: | embryos show strong tissue damage |
| L4: | embryos are completely disintegrated |
| Nec1: | individual necrotic cells (darkened areas in brightfield) |
| Nec2: | many necrotic cells (darkened areas in brightfield) |
|  |  |
| **Developmental toxicity** | |
| D1: | developmental delay (slow development) |
| D2: | developmental defect (malformations) |
|  |  |
| **Cardiotoxicity** | |
| C1: | reduced circulation or heart edema |
| C2: | reduced circulation plus heart edema |
| C3: | no circulation with or without heart edema |
|  |  |
| **Neurotoxicity** | |
| N0: | normal movement in response to touch |
| N1: | reduced movement in response to touch |
| N2: | no movement in response to touch |
|  |  |
| **Overall toxicity (combination of above phenotypes)** | |
| wt: | wild type (no visible phenotype AND normal movement) |
| T1: | embryos that show a phenotype |
| T2: | lethal damage that precludes assessment of other phenotypes (L3, L4, Nec2) |

**Supplementary Figure 7: Effect of the substitution of amino acids with alanine of endogenous α_1_AT peptide from human hemofiltrate on ADP-ribosylation of Gαi in PT-treated CHO-K1 cells. (a-c)** PT (10 ng/ml) and 100 µM α_1_AT HF peptide, 100 µM α_1_AT HF peptide P1-11, 100 µM α_1_AT or the respective amount of solvent (H_2_O) were added directly to CHO-K1 cells in FCS-free medium and incubated for 4 h at 37 °C. For further control cells were left untreated. After the 4 h incubation, cell lysates were generated and Gαi, which had not been ADP-ribosylated during the initial intoxication living cells with PT, was ADP-ribosylated and biotin-labeled via the subsequent incubation of the cell lysates with recombinant PTS1 and biotin-labeled NAD^+^. Next, the biotin-labeled Gαi was detected via Western blot, while Hsp90 served as a control for equal protein loading. The bar graph (a) shows the quantification of Western blot signals from three independent experiments, while (b, c) shows the results of a representative experiment. The intensity values of the bar graph are given as x-fold of the untreated control (Con), normalized to Hsp90, mean ± SEM (n = 5-30 values from eight independent experiments). Significance was tested using one-way ANOVA followed by Dunnett’s multiple comparison test and refers to samples treated with PT only (*p < 0.1, **p < 0.01, ***p < 0.001, ****p < 0.0001, ns not significant).
